# Scavenging Intracellular ROS Attenuates *p*-Cresyl Sulfate-Triggered Osteogenesis through MAPK Signaling Pathway and NF-κB Activation in Human Arterial Smooth Muscle Cells

**DOI:** 10.3390/toxins12080472

**Published:** 2020-07-24

**Authors:** Jia-Feng Chang, Chih-Yu Hsieh, Jian-Chiun Liou, Shih-Hao Liu, Chi-Feng Hung, Kuo-Cheng Lu, Chih-Cheng Lin, Chang-Chin Wu, Shuk-Man Ka, Li-Li Wen, Mai-Szu Wu, Cai-Mei Zheng, Wen-Chin Ko

**Affiliations:** 1 Division of Nephrology, Department of Internal Medicine, En Chu Kong Hospital, New Taipei City 237, Taiwan; cjf6699@gmail.com; 2 Department of Pathology, Tri-Service General Hospital, National Defense Medical Center, Taipei 114, Taiwan; fish37435@hotmail.com; 3 Graduate Institute of Aerospace and Undersea Medicine, Academy of Medicine, National Defense Medical Center, Taipei 114, Taiwan; mariaka@ndmctsgh.edu.tw; 4 Department of Nursing, Yuanpei University of Medical Technology, Hsinchu 300, Taiwan; 5 Renal Care Joint Foundation, New Taipei City 220, Taiwan; 6 Division of Nephrology, Department of Internal Medicine, Taipei Medical University-Shuang Ho Hospital, New Taipei City 235, Taiwan; maiszuwu@gmail.com (M.-S.W.); 11044@s.tmu.edu.tw (C.-M.Z.); 7 School of Biomedical Engineering, Taipei Medical University, Taipei 110, Taiwan; jcliou@tmu.edu.tw; 8 Division of Pathology, En-Chu-Kong Hospital, New Taipei City 237, Taiwan; 01393@km.eck.org.tw; 9 School of Medicine, Fu Jen Catholic University, New Taipei City 242, Taiwan; 054317@mail.fju.edu.tw; 10 Division of Nephrology, Department of Medicine, Taipei Tzu Chi Hospital, Buddhist Tzu Chi Medical Foundation, New Taipei City 231, Taiwan; Kuochenglu@gmail.com; 11 Department of Biotechnology and Pharmaceutical, Yuanpei University, Hsinchu 300, Taiwan; lcc@mail.ypu.edu.tw; 12 Department of Biomedical Engineering, Yuanpei University of Medical Technology, Hsinchu 300, Taiwan; Dtorth65@yahoo.com.tw; 13 Department of Orthopaedic Surgery, En-Chu-Kong Hospital, New Taipei City 237, Taiwan; 14 Department of Clinical Laboratory, En Chu Kong Hospital, New Taipei City 237, Taiwan; eckwen@yahoo.com; 15 Division of Nephrology, Department of Internal Medicine, School of Medicine, College of Medicine, Taipei Medical University, Taipei 110, Taiwan; 16 Division of Cardiac Electrophysiology, Department of Cardiovascular Center, Cathay General Hospital, Taipei 106, Taiwan

**Keywords:** alkaline phosphatase, c-Jun N-terminal kinase, extracellular signal-regulated kinase, mitogen-activated protein kinase, nuclear factor-κB; osteogenesis, p-Cresyl Sulfate, reactive oxygen species, Runt-related transcription factor 2, uremic vascular calcification

## Abstract

Osteogenesis in human arterial smooth muscle cell (HASMC) is a key feature of uremic vascular calcification (UVC). Concerning pro-oxidant properties of *p*-cresyl sulfate (PCS), the therapeutic effect of reactive oxygen species (ROS) scavenger on PCS triggered inflammatory signaling transduction in osteogenesis was investigated in this translational research. Based on severity level of chronic kidney disease (CKD), arterial specimens with immunohistochemistry stain were quantitatively analyzed for UVC, oxidative injury and osteogenesis along with PCS concentrations. To mimic human UVC, HASMC model was used to explore whether PCS-induced ROS could trigger mitogen-activated protein kinase (MAPK) pathways with nuclear factor-κB (NF-κB) translocation that drive context-specific gene/protein expression, including Runt-related transcription factor 2 (*Runx2*) and alkaline phosphatase (ALP). In parallel with PCS accumulation, CKD arteries corresponded with UVC severity, oxidative DNA damage (8-hydroxy-2′-deoxyguanosine), *Runx2* and ALP. PCS directly phosphorylated extracellular signal-regulated kinase (ERK)/c-Jun N-terminal kinase (JNK)/P38 (pERK/pJNK/pP38) and modulated NF-κB translocation to promote expressions of *Runx2* and ALP in HASMC. Notably, intracellular ROS scavenger attenuated pERK signaling cascade and downstream osteogenic differentiation. Collectively, our data demonstrate PCS induces osteogenesis through triggering intracellular ROS, pERK/pJNK/pP38 MAPK pathways and NF-κB translocation to drive *Runx2* and ALP expressions, culminating in UVC. Beyond mineral dysregulation, osteocytic conversion in HASMC could be the stimulation of PCS. Thus PCS may act as a pro-osteogenic and pro-calcific toxin. From the perspective of translational medicine, PCS and intracellular ROS could serve as potential therapeutic targets for UVC in CKD patients.

## 1. Introduction

Cardiovascular (CV) disease remains the most prevalent cause of death in patients with chronic kidney disease (CKD) to date, and uremic vascular calcification (UVC) is at the heart of the matter [1,2,3]. Chronic mineral dysregulation in CKD patients promotes UVC and culminates in premature CV death, even in the early stages of the disease [4,5,6]. Beyond careful attention to disturbances of calcium-phosphate (Ca-P) and vitamin D metabolism, there is an urgent need to explore new therapeutic targets for the intricate mechanism of UVC in clinical practice [7].

The representative protein-bound uremic toxins (PBUT), *p*-cresyl sulfate (PCS) and indoxyl sulfate (IS), exert pro-oxidant and pro-inflammatory effects on diverse cell and organ systems [8,9] PCS mainly presents as a protein-bound form in the blood circulation, and this study is focusing on the free form of PCS [10]. Experimental data from cell and animal models suggest that reactive oxygen species (ROS) and chronic sustained inflammation resting from circulating PTUB are hallmarks of UVC and capable of pivotal inducers of osteogenesis in human arterial smooth muscle cell (HASMC) [11]. Emerging evidence also indicates UVC is an active, HASMC-mediated process resembling osteogenesis in bone rather than passive Ca-P precipitation [12]. Apoptotic bodies derived from HASMCs may also act as crystal nucleation for calcium deposition [13]. The underlying mechanism involved in PTUB have been attributed by various signaling pathways including ROS/c-Jun N-terminal kinase (JNK)/P38/mitogen-activated protein kinases (MAPK) [14], aryl hydrocarbon receptor (AhR)/nuclear factor kappa B (NFκB)/ MAPK [15], and ROS-activated NF-κB/extracellular signal-regulated kinases (ERK)/ JNK cascades [16]. In vitro, IS accentuated Pi-induced VSMC calcification and osteogenic transition through increasing Pit-1 expression [17] and oxidative stress [17]. Despite previous documented implications, the pro-calcific effect of solely PCS on the actively cell-regulated process of osteogenic conversion in HASMC remains elusive, resting in a lack of in-depth understanding of the molecular mechanism and therapeutic targets, we aimed to investigate the therapeutic effect of intracellular ROS scavenger on PCS triggered inflammatory signaling transduction in HASMC osteogenesis.

## 2. Results

### 2.1. Circulating Levels of PCS/ IS were CKD Severity-Dependent and Positively Correlated with Ca-P Product and Alkaline Phosphatase (ALP), Suggesting that PCS is a Long-Neglected Mineralization Inducer in UVC

Twenty-seven human amputation specimens were used in the experiments: controls (*n* = 9), mild-to-moderate CKD (*n* = 9), and severe CKD (*n* = 9). Comparisons of bio-clinical characteristics among three study groups of normal controls, mild-to-moderate and severe CKD were summarized in the Table 1. The background bio-demographic characteristics were generally similar among three study groups, except the renal function related profiles. Our data demonstrated there is a statistically significant difference in aortic arch calcification (AAC) grade score, circulating ALP, phosphate, Ca-P product, PCS and IS between study groups of normal controls, mild-to-moderate and severe CKD. Circulating levels of PCS and IS were CKD severity-dependent and positively correlated with AAC (Spearman’s correlation coefficients were 0.40 and 0.35, respectively). Above results demonstrated PCS and IS are associated with estimated glomerular filtration rate (eGFR) decline, mineral dysregulation and vascular calcification, suggesting that PCS and IS may act as pro-calcific toxins.

### 2.2. Effects of CKD Severity-Dependent Vascular Oxidative Injury on HASMC Osteogenesis in Human Arteries

**Figure 1:**
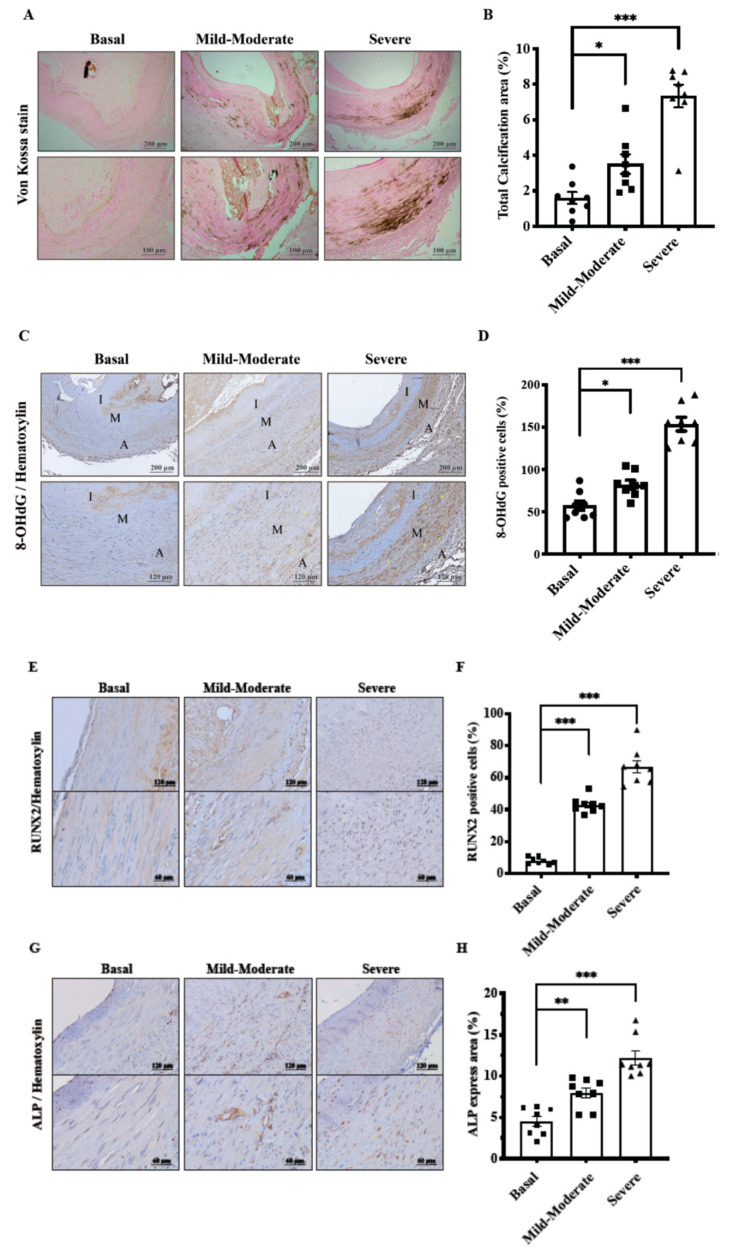
Effects of CKD severity-dependent vascular oxidative injury on HASMC osteogenesis in human arteries. Muscular arteries were classified into three study groups of normal controls, mild-to-moderate and severe CKD. Control groups, eGFR ≥ 60 mL/min; Mild-to-moderate CKD, 15 mL/min < eGFR ≤ 60 mL/min; Severe CKD, eGFR ≤ 15 mL/min. (**A**,**B**) Calcium deposits in UVC areas were examined using von Kossa stain. (**C**,**D**) Vascular oxidative injury was examined by immunohistochemical staining of 8-OHdG in nuclei of HASMCs. (**E**–**H**) Osteogenic conversion of HASMC was identified with Rux2 and ALP. Twenty-seven human samples were used in the experiments: controls (*n* = 9); mild-to-moderate CKD (*n* = 9); severe CKD (*n* = 9). For image analyses, three random high-resolution fields were obtained in each sample. Scale bars in the upper panels are 200 μm and 100 μm in the lower panels. Quantitative analyses for staining of von Kossa, 8-OHdG, Rux2 and ALP were performed using ImageJ software. Data are expressed as mean ± SD. Data were analyzed with ANOVA (Dunnett’s multiple comparisons test). * *p* < 0.05; ** *p* < 0.01; *** *p* < 0.001. 8-OHdG, 8-hydroxy-2’-deoxyguanosine; A, adventitia; ALP, alkaline phosphatase; CKD, chronic kidney disease; I, intima; M, media; *Runx2*, runt-related transcription factor 2; UVC, uremic vascular calcification.

Arteries from control group appeared histologically intact, whereas CKD arteries exhibited progressive calcium deposition identified by von Kossa staining method (Figure 1A). As expected, the quantified UVC area was in parallel with the escalating severity of CKD (Figure 1B). Further, UVC is reminiscent of vascular oxidative injury in response to chronic stimuli of uremic milieu. Through immunohistochemical (IHC) staining methods, the indicator of ROS-mediated DNA damage, 8-hydroxy-2’-deoxyguanosine (8-OHdG), was in accordance with CKD staging (Figure 1C,D).

Under harsh environmental condition of oxidative stress and mineral dysregulation, maladaptive change of HASMCs could initially prolong survival, yet timately resting in progressive UVC and CV events. Our data indicated CKD severity-dependent *Runx2* expression corresponded with positive staining of UVC and 8-OHdG, indicative of osteogenic differentiation *(*Figure 1E,F). *As shown in* Figure 1G,H, the incremental expression of ALP was in line with CKD staging. Current results demonstrate that oxidative stress in the uremic milieu triggers active HASMC osteogenic differentiation, resulting in catastrophic UVC.

### 2.3. PCS Inhibits Cell Viability of HASMC in a Time- and Dose-Dependent Manner

**Figure 2:**
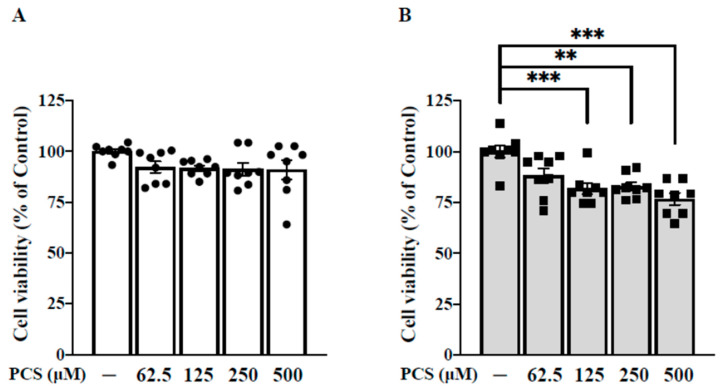
PCS inhibits cell viability of HASMC in a time- and dose-dependent manner. (**A**) HASMCs were treated with various concentrations of PCS (0, 62.5, 125, 250, and 500 μM) for 24 h. (**B**) HASMCs were treated with various concentrations of PCS (0, 62.5, 125, 250, and 500 μM) for 48 h. Cell viability was determined using the colorimetric Alamar Blue assay. Data were expressed as mean ± standard deviation. Each experiment was replicated for three times. Data were analyzed with ANOVA (Dunnett’s multiple comparisons test). ** *p* < 0.01, and *** *p* < 0.001 indicated significant differences from the control group. HASMC—human arterial smooth muscle cell; PCS—*p*-cresyl sulfate.

To extend the above findings of PBUT toxicity to basic research, cell viability was determined by the colorimetric Alamar Blue assay in PCS-treated HASMC cell model with a 24–48 h time window. As shown in Figure 2A, the quantitative analysis elucidated HASMCs exposed to various concentrations of PCS (62.5, 125, 250, and 500 μM) for 24 h did not exerted significant decrease in cell viability compared with the basal group (0 μM PCS; 0.1% DMSO). As shown in Figure 2B, the quantitative analysis indicated higher concentrations of PCS (125, 250, and 500 μM) with an incubation period of 48 h induced significant cell death compared with the basal group (0 μM PCS; 0.1% DMSO). Above results demonstrated PCS inhibited cell viability of HASMC in a time- and dose-dependent manner. It is noteworthy that an incubation period of 48 h incurs significant cell death. To prevent PCS-induced cell death, an incubation period within 24 h was optimal to monitor active HASMC-regulated osteogenic differentiation.

### 2.4. PCS Triggers Phosphorylation of the MAPK Family Members (JNK, ERK and P38) in HASMC: Intracellular ROS Scavenger Inhibits PCS-Induced pERK MAPK Pathway

**Figure 3:**
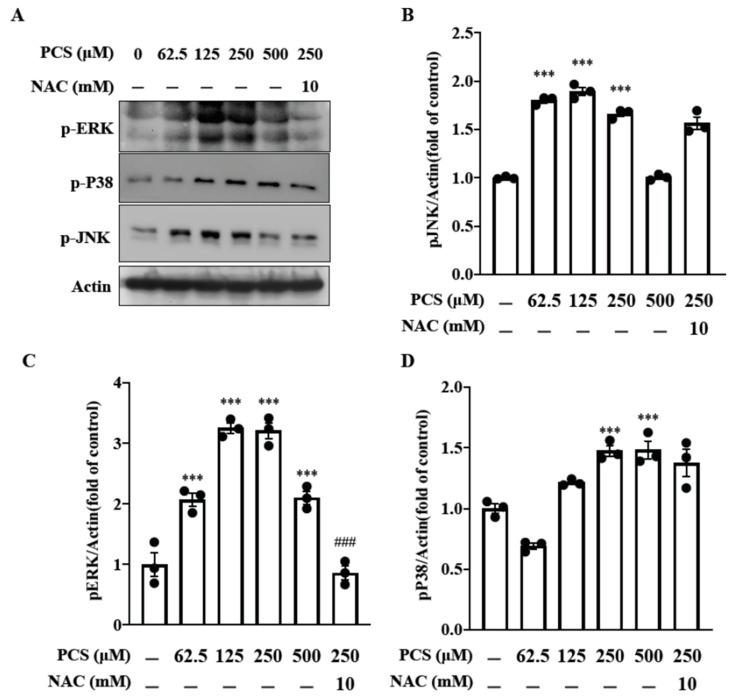
Effect of PCS on phosphorylation of the MAPK family members (JNK, ERK and P38): intracellular ROS scavenger NAC inhibits PCS-induced pERK MAPK pathway in HASMC. (**A**) HASMC cultures were exposed to various concentrations of PCS (0, 62.5, 125, 250, and 500 μM) for 24 h. Then HASMCs were lysed and subjected to western blotting using specific antibodies against phosphorylated and total isoforms of JNK (pJNK), ERK (pERK) and P38 (pP38). Bands were visualized using enhanced chemiluminescence detection reagent and the band intensities were quantified by densitometric analysis. (**B**–**D**) Quantification analyses for pJNK, pERK and pP38 were expressed in fold change: the ratio of the density of phosphorylated isoform to that of total isoform at indicated concentrations. Data were expressed as mean ± standard deviation. Each experiment was replicated for three times. Data were analyzed with ANOVA (Dunnett’s multiple comparisons test). *** *p* < 0.001 indicated significant differences from the control group (0 μM). ### *p* < 0.001 indicated significant differences from the treatment group (250 μM). HASMC—human arterial smooth muscle cell; NAC—N-acetyl-l-cysteine; PCS—*p*-cresyl sulfate.

The pro-oxidant and pro-inflammatory effects of PTUB are related to the intricate signaling cascade of ROS/ MAPK pathways in diverse cell models [14,15,16]. Nonetheless, data about therapeutic targeting PCS induced MAPK activation in HASMC are still lacking. To investigate the therapeutic effect of intracellular ROS scavenger on PCS-induced MAPK activation in HASMCs, phosphorylation of JNK/ ERK/ P38 (pJNK/ pERK/ pP38) were determined at different concentrations PCS (0, 62.5, 125, 250, and 500 μM) with or without the treatment of N-acetyl-L-cysteine (NAC) (10 mM). Our findings demonstrate PCS induces expressions of pJNK/ pERK/ pP38 in a dose-dependent manner (Figure 3). Furthermore, NAC inhibits PCS-induced pERK MAPK activation but not pJNK/ pP38 signaling transduction in HASMC. Current data demonstrate that PCS-induced intracellular ROS is a key messenger to activate downstream pERK MAPK pathway in HASMC. Therefore, PCS and intracellular ROS may serve as the therapeutic targets.

### 2.5. PCS Triggers Translocation of NF-κB From the Cytoplasm to the Nucleus in HASMC

**Figure 4:**
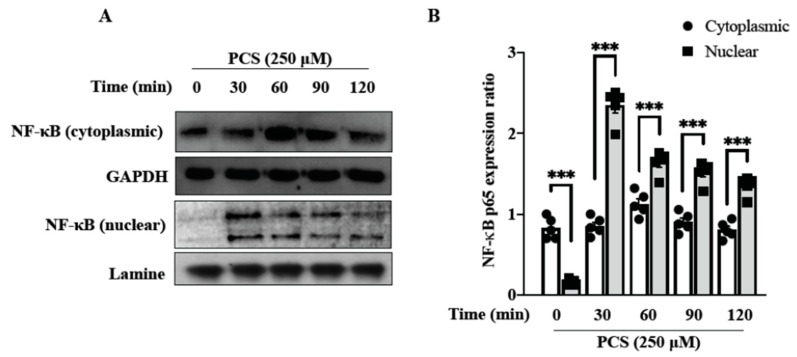
Western blotting of PCS induced nuclear translocation of NF-κB in HASMC. (**A**) HASMC cultures were exposed to PCS (250 μM) for indicated times. After PCS treatment, cell lysates were separated into cytosolic and nuclear fractions, then subjected to western blotting for NF-κB. Lamin B was used as an internal control for nuclear fraction and GAPDH was used as an internal control for cytosolic fraction. (**B**) Densities of the western blotting bands were analyzed with GraphPad Prism 8 (GraphPad Software, Inc., San Diego, CA, USA). Data were expressed as mean ± standard deviation. Each experiment was replicated for three times. Data were analyzed with ANOVA (Dunnett’s multiple comparisons test). *** *p* < 0.001 indicated significant differences between the cytosolic and nuclear fractions. HASMC—human arterial smooth muscle cell; NF-κB—nuclear factor kappa B; PCS—*p*-cresyl sulfate.

NF-κB is a ubiquitous transcription factor that drives context-specific gene expression, and the MAPK signaling pathway is essential in regulating IS-induced cellar stress responses [15]. In proximal tubular cells, IS enhances the expression of NF-κB, which plays a pivotal role in ROS-activated ERK/JNK pathways [16]. To investigate whether NF-κB was involved in PCS-induced activation of MAPK pathways, HASMC cultures were treated with PCS. Western blotting was then conducted to detect translocation of NF-κB from the cytoplasm to the nucleus (Figure 4). After PCS (250 μM) treatment for indicated times (0, 30, 60, 90, 120 min), cell lysates were then separated into cytosol fraction and nuclear fraction. Western blotting results for NF-κB in each fraction showed that PCS-activated NF-κB expression increased in the nucleus and reduced in the cytoplasm. Our result demonstrated translocation of NF-κB from the cytoplasm to the nucleus in HASMC, indicating that NF-κB is responsible for the activation of MAPK pathways.

### 2.6. PCS-Induced Intracellular ROS Triggers Osteogenic Conversion of HASMC, and ROS Scavenger NAC Inhibits PCS-Induced Osteogenic Transcription Factor Runx2 and Osteoblast-Specific Protein ALP

In CKD patients, circulating level of IS was associated with ROS accumation and aortic calcification [11,18]. Experimental data from animal models unveiled IS induced aortic calcification in hypertensive rats [19]. Despite previous documented implications, the pro-oxidant and pro-calcific effect of solely PCS on the actively cell-regulated process of osteogenic conversion in HASMC remains elusive.

**Figure 5:**
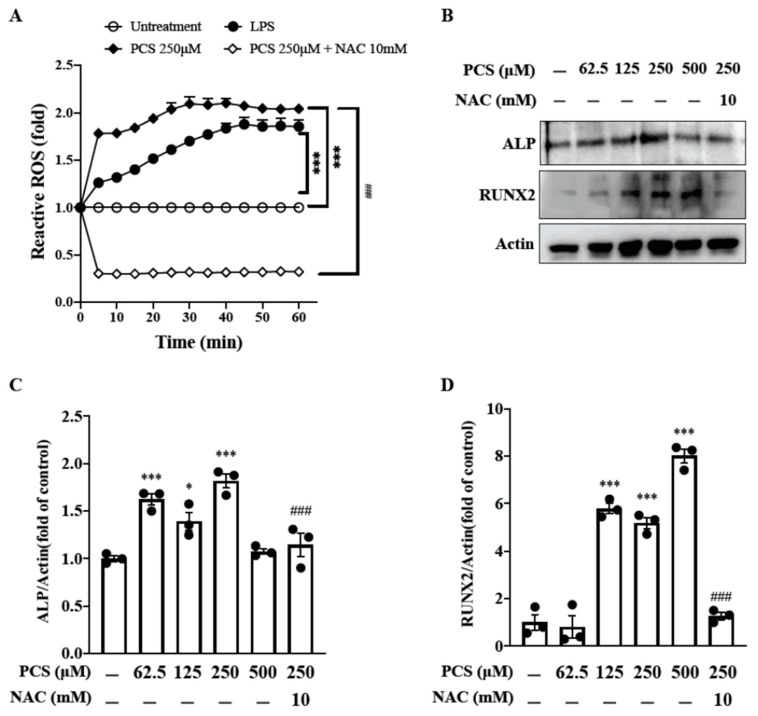
Therapeutic effects of intracellular ROS scavenger NAC on PCS-induced activations of ROS, osteogenic transcription factor *Runx2* and osteoblast-specific protein ALP in HASMC. (**A**) PCS-induced intracellular ROS production was measured by detecting the fluorescence intensity of 2′, 7′-dichlorofluorescein, the oxidation product of 2′, 7′-dichlorofluorescein diacetate (Molecular Probes, Eugene, OR, USA). The cells were pretreated with NAC 10 mM or PBS for 30 min and then incubated with 2′,7′-dichlorofluorescein diacetate (2 mM) for an additional 60 min. The cells were then stimulated with 5 μg/mL LPS, 250 μM PCS or 0.1% DMSO for the indicated time. (**B**) HASMC cultures were exposed to various concentrations of PCS (0, 62.5, 125, 250, and 500 μM) for 24 h. Then HASMCs were lysed and subjected to western blotting using specific antibodies against *Runx2* and ALP. Bands were visualized using enhanced chemiluminescence detection reagent and the band intensities were quantified by densitometric analysis. (**C**,**D**) Quantification analyses for *Runx2* and ALP were expressed in fold change. Data were expressed as mean ± standard deviation. Each experiment was replicated for three times. Data were analyzed with ANOVA (Dunnett’s multiple comparisons test). * *p* < 0.05, and *** *p* < 0.001 indicated significant differences from the control group (0 μM PCS; 0.1% DMSO). ### *p* < 0.001 indicated significant differences from the treatment group (250 μM). ALP—alkaline phosphatase; HASMC—human arterial smooth muscle cell; LPS—lipopolysaccharides; NAC—N-acetyl-l-cysteine; PCS—*p*-cresyl sulfate; *Runx2*—Runt-related transcription factor 2.

Thus we used the ROS-specific indicator 2′,7′-dichlorodihydrofluorescein diacetate to detect PCS-induced intracellular ROS production. HASMCs were pretreated with NAC 10 mM or PBS for 30 min and then incubated with 2′,7′-dichlorofluorescein diacetate (2 mM) for an additional 60 min. The cells were then stimulated with 5 μg/mL lipopolysaccharides (LPS) (as a positive control), 250 μM PCS or 0.1% DMSO for the indicated time. Our data showed that NAC significantly reduced PCS-induced intracellular ROS in HASMCs. To investigate the therapeutic effect of intracellular ROS scavenger on PCS-triggered osteogenic conversion of HASMC, osteogenic transcription factor *Runx2* and bone formation marker ALP were determined at different concentrations PCS (0, 62.5, 125, 250, and 500 μM) with or without the treatment of NAC (10 mM). Our findings demonstrate PCS accentuates expressions of intracellular ROS, transcription factor *Runx2* and osteoblast-specific protein ALP (Figure 5). Notably, NAC inhibits PCS-induced activation of *Runx2* and ALP in HASMC, suggesting NAC turns off transcription factor-regulated osteogenic gene expression. Current data demonstrate that PCS-induced intracellular ROS may serve as a therapeutic target to disrupt downstream signaling transduction pathways of osteogenic conversion in HASMC.

## 3. Discussion

UVC has traditionally been viewed as a passive process that is associated with hyper- phosphatemia, calcium-based therapies, disturbance of mineral metabolism, hyperparathyroidism and elevated circulating levels of Ca-P product. Nowadays, UVC is proven to be an actively HASMC-regulated osteogenesis associated with various bone-specific proteins in bone mineralization [20]. According to recent data from cell-based and animal models, elevated extracellular Ca-P and PBUT concentrations promote UVC synergistically, suggesting that PCS may act as a pro-osteogenic and pro-calcific toxin [11]. PCS-triggered pro-calcifying mechanism plays a critical role in osteogenesis of HASMC, making this area an active focus of research. Considering the UVC treatment in CKD patients, the identification of therapeutic targets which can mitigate or even reverse the progression is an important step forward. To gain a more comprehensive insight into PCS-driven inflammatory signaling pathways in UVC, the human model of arterial tissue sections and HASMC cell model were used in this translational research. Several important issues deserve further discussion.

UVC is reminiscent of oxidative DNA damages in HASMC due to chronic stimuli of PBUT [11]. Our human models of medium-sized muscular arteries exhibited higher expressions of 8-OHdG, corresponding with CKD severity-dependent UVC (Figure 1). Further, *Runx2* is upregulated in the osteocytic conversion of HASMC and serves as an essential transcription factor driving downstream ALP expression. It is reported that ALP is a byproduct of osteoblast activity in the active reprogramming process of HASMC [3]. As expected, our data also demonstrated CKD severity-dependent *Runx2* expression corresponded with positive staining of ALP and UVC, indicative of osteogenic differentiation. Under the harsh milieu of PTUB-induced oxidative stress, maladaptive change of HASMCs could initially prolong survival, yet ultimately resulting in progressive UVC and CV events [21]. This shows ROS may act as a key regulator of osteoblast differentiation and trigger downstream *Runx2* and ALP. Barreto et al. reported serum concentrations of IS were correlated with aortic calcification and CV death in CKD patients [18]. scarce, suggesting that PCS is a long-neglected mineralization inducer in UVC.

Although our human data revealed higher circulating levels of PCS were associated with higher pro-calcific stress, the mechanism and therapeutic targets of PCS triggered inflammatory signaling transduction in HASMC osteogenesis were still unclear. From the perspective of translational medicine, we outreached above clinical findings of PBUT toxicity to basic research. Given that osteocytic conversion is a maladaptive change of HASMCs to prolong survival in response to cytotoxic stimulation, the time course and dose effect of PCS shod be tested to avoid cell death. Thus cell viability was determined by various doses of PCS using the colorimetric Alamar Blue assay in HASMC cell model with a 24–48 h time window. Our data demonstrated PCS inhibited HASMC viability in a time- and dose-dependent manner (Figure 2). To prevent pro-death effect of PCS, an incubation period within 24 h was optimal to monitor PCS-regulated osteogenic differentiation in HASMC.

We previously reported that intracellular sources of ROS induced by PCS are multifaceted, including NADPH oxidase and mitochondria [8]. Our prior findings elucidated single inhibitors for NADPH oxidase-derived ROS and mitochondria-targeted superoxide could not abrogate downstream inflammatory responses to attenuate cell damages, reflecting the underlying signaling transduction network is intricate. In the current study, our data demonstrate intracellular ROS scavenger NAC inhibits PCS-induced pERK MAPK activation but not pJNK/pP38 signaling pathways in HASMC. Upon cell stimulation, the nuclear localization signal on NF-κB becomes exposed and the protein translocated to the nucleus [22]. NF-κB nuclear translocation turns on transcription factors and activates specific gene/protein expression. Shimizu et al. reported that IS enhances the expression of NF-κB, which is one of the most important regulators for ROS-activated ERK/JNK pathways in proximal tubular cells [16]. Corresponding to the prior research, our results unveil translocation of NF-κB from the cytoplasm to the nucleus in HASMC, indicating that NF-κB is responsible for the activation of MAPK pathways (Figure 4).

**Figure 6:**
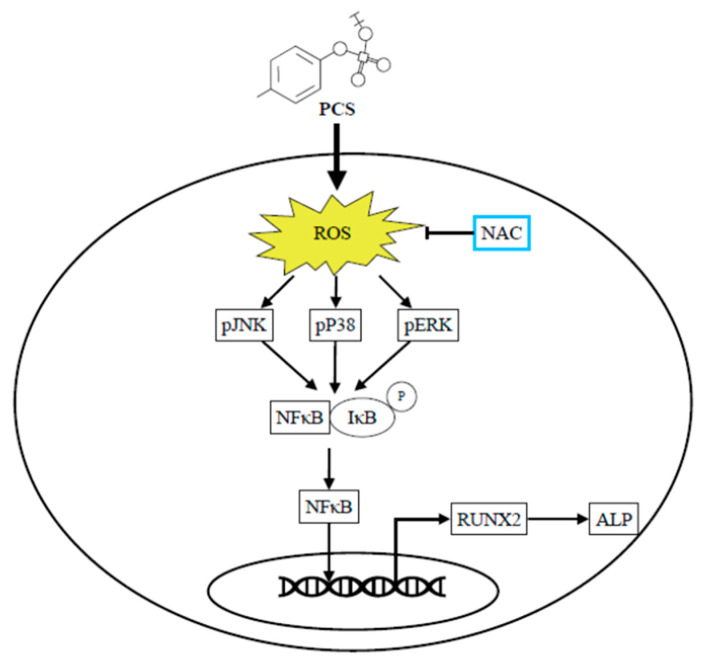
Scavenging intracellular ROS attenuates PCS triggered osteogenesis through pERK signaling pathway and NF-κB activation in HASMC. The exposure to PCS directly trigger intracellular ROS, phosphorylation of ERK/p38/pJNK and nuclear translocation of NF-κB that drive context-specific gene/protein expression (*Runx2* and ALP) in HASMC. Notably, intracellular ROS scavenger NAC attenuated pERK MAPK cascade and downstream osteogenic differentiation. Thus pERK/NF-κB pathway may act as the key signaling transduction in the mechanism of HASMC osteogenesis. In light of this, PCS may act as a pro-osteogenic and pro-calcific toxin. From the perspective of translation to humans, PCS and intracellular ROS serve as potential therapeutic targets for uremic vascular calcification in CKD patients. ALP, alkaline phosphatase; CKD, chronic kidney disease; ERK, extracellular signal-regulated kinase; HASMC, human arterial smooth muscle cells; JKK, c-Jun N-terminal kinase; MAPK, mitogen-activated protein kinase; NAC, N-acetyl-L-cysteine; NF-κB, nuclear factor-κB; PCS, *p*-cresyl sulfate; p, phosphorylation; ROS, reactive oxygen species; *Runx2*, runt-related transcription factor 2.

A growing body of evidence from in vitro and in vivo experiments suggests that IS may directly impact the development of UVC. Muteliefu et al. demonstrated IS triggered ROS production and the expression of NADPH oxidases (Nox4), *Runx2*, ALP and osteopontin in HASMCs [9]. An antioxidant of NADPH oxidase inhibitor and knockdown of Nox4 using small interfering RNA (siRNA) could inhibit such ROS generation and expressions of *Runx2* and ALP. Wu et al. also reported IS promotes osteocytic conversion and matrix mineralization through activating expressions of Pit-1 and JNK pathway in primary human umbilical vein smooth muscle cells [17]. Adijiang et al. demonstrated Dahl salt-sensitive hypertensive rats fed with IS exert aortic wall thickening and aortic calcification through the upregulation of osteoblast-specific proteins [19]. To date, literature is scarce regarding the pro-calcific effect of PCS on the osteogenic conversion of HASMC without other mineralization inducers in cell culture. Furthermore, ROS is a well-known driver of UVC [20]. A recent study demonstrated PCS can directly trigger UVC in aorta/ peripheral arteries of CKD rats through inducing insulin resistance and hyperglycemia that activate the coagulation pathways and the acute-phase reaction signaling in the arterial wall [23]. In the process of UVC, HASMCs undergo further maladaptive osteocytic conversion and express markers normally restricted to bone, such as the osteogenic transcription factors *Runx2* and the mineralization regulating proteins ALP [3]. Accordingly this inspires us to explore whether solely the stimulation of PCS triggers osteogenic conversion of HASMC through ROS-modulated pERK kinase activity and NF-κB nuclear translocation to turn on osteogenesis. As expected, PCS accentuates expressions of intracellular ROS, transcription factor *Runx2* and osteoblast-specific protein ALP (Figure 5). We provide a brand-new idea that osteogenic transition of HASMC could be solely the stimulation of PCS. osteogenic transition of HASMC could be solely the stimulation of PCS. Of prime importance is NAC treatment attenuates PCS-induced activation of *Runx2* and ALP in HASMC. Specifically, transcription factor-regulated osteogenesis can be turned off through eradicating intracellular ROS. In light of this, PCS and PCS-induced intracellular ROS may serve as therapeutic targets to abrogate downstream signaling transduction of osteocytic conversion in HASMC (Figure 6).

Our study has several limitations. To begin with, our patient sample size was relatively small and thereby the results should be interpreted with caution. Next, the toxic effects of IS on the HASMC were not examined for comparison in our study. In addition, our cell model could be only used to explain the patho-mechanism of acute exposure of PCS.

## 4. Conclusions

UVC is an inevitable complication in CKD patients to raise the risk of CV death. Our data demonstrate PCS induces osteogenesis through triggering intracellular intracellular ROS, pERK MAPK pathways and nuclear translocation of NF-κB to enhance downstream *Runx2* and ALP expressions. Beyond mineral dysregulation, osteogenic transition in HASMC can be solely the stimulation of PCS, reflecting PCS is a pro-calcific toxin. Thus PCS and PCS-induced intracellular ROS serve as potential therapeutic targets for UVC in CKD patients.

## 5. Materials and Methods

### 5.1. IHC Staining and Image Analyses

Tissue sections of human arterial rings were evaluated quantitatively and qualitatively. The histological characterizations were assessed by hematoxylin and eosin stain. A Nikon Digital Camera Microscope (Nikon, Tokyo, Japan) was used for image capture. For tissue staining of arterial ring sections, we generally followed the introductions of the manufaculturer’s protocol: (1) von Kossa staining (CVK-1-IFU, ScyTek Laboratories, Logan, UT, USA.) to detect calcified lesions; (2) 8-hydroxy-2’-deoxyguanosine (8-OHdG) (Bs-1278R, Bioss Antibodies, Woburn, MA, USA. 1:200 dilution) for oxidative stress related vascular injury; (3) *RUNX2* (Sc-101145, Biotechnology, Santa Cruz, CA, USA. 1:200 dilution) as an osteoblast-specific transcription factor; (4) ALP (Ab108337, Abcam, Cambridge, UK. 1:200 dilution) as a bone formation marker. The details of the quantitative method for IHC images were proposed previously [3]. In brief, three random high-resolution fields in each specimen were used for quantitative analyses via ImageJ version 1.48v image analysis software (National Institutes of Health, Bethesda, MD, USA).

### 5.2. Reagents and Chemicals

PCS was purchased from Alsachim (Illkirch-Graffenstaden, France). NAC was purchased from Sigma-Aldrich (St. Louis, MO, USA). Antibodies against NF-κB p65, p-ERK, p-JNK and p-P38 were obtained from Cell Signaling Technology (Beverly, MA, USA). Antibodies against ALP were obtained from Abcam (Cambridge, UK). Anti-*RUNX2* and other antibodies can be obtained from Santa Cruz Biotechnology (Santa Cruz, CA, USA). The Alamar Blue assay kit was purchased from Thermo Fisher Scientific (Waltham, MA, USA).

### 5.3. Cell and Treatments

The primary aortic smooth muscle cells (HASMC; ATCC PCS-100-012) were purchased from the American Type Culture Collection (Rockville, MD, USA) and cultured in the vascular cell basal medium (ATCC PCS-100-030) with a vascular smooth muscle cell growth kit (ATCC PCS-100-042) at 37 °C in a 5% CO_2_ incubator. The passage number of HASMC cells in the experiments was P7-P9.

### 5.4. Cell Viability Assay

Cell viability was assessed by Alamar Blue assay (Thermo Fisher Scientific, Waltham, MA, USA). In short, cells were seeded into 96-well plate at a density of 8000 cells/well and stabilized at 37 °C in 5% CO_2_ for 24 h. Cells were incubated for 24 h or 48 h with or without PCS (62.5, 125, 250, 500 μM). Cell viability was determined using colorimetric Alamar Blue assay according to the direction of manufaculturer. Each experiment was replicated for three times.

### 5.5. Sequential Fractionation and Isolation of Subcellar Proteins

Samples were kept on ice throughout the procedure and all centrifugations were done at 4 °C. Cells were transferred from 10 cm plates into 500 μL fractionation buffer. After incubation on ice for 15 min, the cell suspension was passed through a 26 gauge needle for 10 times to lyse all cells with 1 mL syringe. Samples were centrifuged at 720× *g* (3000 rpm) for 5 min. The supernatant was transferred into a fresh tube for further extraction of cytoplasm, membrane and mitochondria. The nuclear pellet was washed with 500 μL of fractionation buffer, dispersed using a pipette, and passed through a 25 gauge needle 10 times. Following a 10 min centrifugation, the supernatant was discarded. The pellet that contains nuclei was kept and resuspended in TBS with 0.1% SDS. The suspension was sonicated briefly to shear genomic DNA and homogenize the lysate.

### 5.6. Detection of Intracellular ROS Production

Intracellular ROS production was measured by detecting the fluorescence intensity of 2′, 7′-dichlorofluorescein, the oxidation product of 2′, 7′-dichlorofluorescein diacetate (Molecular Probes, Eugene, OR, USA) as described previously [24,25]. In brief, 12-well plates (8 × 10^4^ cells/well) with 0.1 mL of culture medium were grown overnight at 37 °C in a 5% CO_2_ incubator. The cells were pretreated with NAC 10 mM or PBS for 30 min and then incubated with 2′,7′-dichlorofluorescein diacetate (2 mM) for an additional 60 min. The cells were then stimulated with 5 μg/mL LPS, 250 μM PCS or 0.1% DMSO for the indicated time.

### 5.7. Measurement of Circulating PCS and IS Levels

The blood samples (50 μL) were pretreated with 1400 μL acetonitrile (ACN) to precipitate proteins, followed by centrifugation at 13,400× *g* for 20 min at 4 °C. Each tube of the supernatant was evaporated using a spin vacuum instrument. Then we redissolved above lyophilized samples in 200 μL 30% ACN aqueous solution with 0.1% formic acid (FA). PCS and IS were analyzed with a tandem mass spectrometry (MS) system (Thermo Finnigan TSQ Quantum tra Mass Spectrometer, Thermo Fisher Scientific Inc., Waltham, MA, USA). The tandem MS system was equipped with a Micro ESI ion source, which was set at 3.0 kV, coupled with an Accela 1250 UHPLC analytical system (Thermo Fisher Scientific Inc., Waltham, MA, USA). The samples containing mixtures of PCS and IS were sequentially injected into the UHPLC via the Accela 1250 autosampler and separated using a Shiseido HPLC CAPCELL PAK C18 MGII column (150 mm × 1.5 mm, 3.0 μm, Tokyo, Japan). The mobile phases were composed of (A) 0.1% (*v*/*v*) FA in water, and (B) 0.1% (*v*/*v*) FA in ACN, with a 250 μL/ min flow rate, and the linear gradient was set as follows: 30% (B) in 2 min, 30–60% (B) in 6 min, 60–98% (B) in 3 min, 98% (B) in 2 min, 98–30% (B) in 0.1 min and 30% (B) in 6.9 min. The detection mode of the MS system was set at an applied voltage of 2.5 kV in the negative ion mode, and the vaporizing and capillary temperatures were set at 300 °C and 350 °C, respectively. The sheath gas and aux gas pressures were set at 35 and 10, respectively, with a collision pressure of 1.5 and a collision energy adjusted to 22 V. We used the multiple reaction monitoring (MRM) scanning mode for quantification: MRM transitions 187 > 80 and 187 > 107 belonging to PCS, and 212 > 80 and 212 > 132 belonging to IS. The Xcalibur software (version 2.2, Thermo-Finnigan Inc., San Jose, CA, USA) was used to acquire the MS spectra and control the mass spectrometer.

### 5.8. Statistics

All data were expressed as the mean ± SD using the GraphPad Prism 8 (GraphPad Software, Inc., San Diego, CA, USA) or SPSS version 22.0 (IBM, Armonk, NY, USA). The ImageJ version 1.48v image analysis software (National Institutes of Health, Bethesda, MD, USA) was used to quantify the area percentage of IHC images. Quantitative data were analyzed with ANOVA (Dunnett’s multiple comparisons test) for three groups. A *p*-value < 0.05 was considered statistically significant for each of the experiments.

## 6. Patients and Bio-Clinical Data

According to the ethical standards of the committee and the Helsinki declaration for human research, our study was approved by the Research Ethics Review Committee of the En Chu Kong Hospital (ECKIRB1050402; Date: 17 August 2016; ECKIRB1071203; Date: 18 April 2019). From December 2016 to January 2020, surgical patients with or without CKD following lower-extremity amputation were enrolled in this study. The underlying diseases included traumatic limb amputation, chronic critical limb ischemia, and non-healing lower extremity ulcers with gangrene, etc. The tissue sections of medium-sized muscular arteries were collected for further investigation. The following bio-clinical and laboratory parameters were obtained for further analysis: age, gender, systolic blood pressure, diastolic blood pressure, blood urea nitrogen (BUN), creatinine (Cr), estimated glomerular filtration rate (eGFR), glucose, sodium, potassium, calcium, phosphate, calcium, calcium-phosphate product, ALP, alanine aminotransferase, PCS and IS. Patients with incomplete data were excluded from the study. Patients with hepatobiliary diseases were excluded in our study after careful chart review. Thus the origins of elevated ALP concentrations were determined to be non-hepatic, such as CKD-mineral bone diseases or skeletal events [26]. Non-hepatic ALP is one of the most important osteoblast marker proteins for bone mineralization, hydrolysis of mineralization inhibitor pyrophosphate, and UVC associated mortality in CKD patients [27]. Twenty-seven patients were included and classified into three groups for final analysis: controls (eGFR > 60 mL/min), mild-to-moderate CKD (15 mL/min < eGFR ≤ 60 mL/min) and severe CKD (eGFR ≤ 15 mL/min).

The standard chest radiograph was taken in posterior-anterior view. A clinician specializing in thoracic radiology and blinded to patient’s clinical data independently reviewed one pre-selected standard chest radiograph obtained from each patient within or as close to the amputation surgery as possible. To determine the severity of AAC detectable on chest radiograph, we modified a simple AAC grading system: grade point 1 (no visible calcification), grade point 2 (small spots of calcification or single thin calcification of the aortic knob), grade point 3 (one or more areas of thick calcification, but ≤50% of the circular area of the aortic knob), and grade point 4 (circular calcification with >50% of circular area of the aortic knob) [28].

## Figures and Tables

**Table 1:** Comparisons of bio-clinical characteristics among study groups of normal controls, mild-to-moderate and severe CKD ^a^.

Variables	Controls (*n* = 9)	Mild-to-Moderate CKD (*n* = 9)	Severe CKD (*n* = 9)	*p*-Value
Age (years)	67.1 ± 17.0	70.4 ± 10.3	61.3 ± 13.9	0.39
Male, *n* (%)	7 (77.8)	6 (66.7)	7 (77.8)	0.84
Systolic blood pressure (mmHg)	131.7 ± 31.3	132.0 ± 24.4	130.0 ± 27.9	0.98
Diastolic blood pressure (mmHg)	75.2 ± 14.1	76.2 ± 15.0	75.1 ± 13.9	0.98
Blood urea nitrogen (mg/dL)	17.7 ± 7.0	46.1 ± 24.0	48.2 ± 25.0	<0.01
Creatinine (mg/dL)	0.8 ± 0.3	2.2 ± 0.6	5.6 ± 2.4	<0.01
eGFR (mL/min)	88.5 ± 20.8	32.9 ± 15.8	12.3 ± 4.5	<0.01
Sodium (mmol/L)	136.2 ± 6.1	136.2 ± 5.4	134.0 ± 2.5	0.55
Potassium (mmol/L)	3.8 ± 5.6	4.0 ± 0.7	4.4 ± 0.7	0.16
Glucose (mg/dL)	146.0 ± 43.6	238.8 ± 92.7	249.1 ± 136.3	0.07
Alanine aminotransferase (IU/L)	33.2 ± 22.8	20.7 ± 10.8	16.8 ± 12.6	0.12
Alkaline phosphatase (IU/L)	128.6 ± 40.8	147.5 ± 12.9	202.5 ± 75.5	0.02
Calcium (mg/dL)	8.9 ± 0.4	8.8 ± 0.7	8.8 ± 1.0	0.95
Phosphate (mg/dL)	3.0 ± 0.4	4.3 ± 0.7	5.6 ± 1.2	<0.01
Calcium-phosphate product ^b^	26.8 ± 2.8	37.9 ± 7.0	48.9 ± 9.2	<0.01
AAC grade score	2.0 ± 1.0	2.3 ± 0.9	3.1 ± 0.6	<0.05
PCS (μM)	6.9 ± 4.8	52.7 ± 20.7	109.0 ± 42.0	<0.01
IS (μM)	5.2 ± 2.8	50.2 ± 35.2	108.5 ± 31.0	<0.01

^a^ Severe CKD, eGFR ≤ 15 mL/min; Mild-to-moderate CKD, 15 mL/min < eGFR ≤ 60 mL/min; Control groups, eGFR ≥ 60 mL/min. ^b^ Calcium-phosphate product—Calcium × Phosphate; Continuous variables were expressed as mean ± SD. Categorical variables are expressed as *n* (%). AAC—Aortic arch calcification; CKD—chronic kidney disease; eGFR—estimated glomerular filtration rate; IS—indoxyl sulfate; PCS—*p*-cresyl Sulfate.

## References

[1] Liabeuf S., Desjardins L., Diouf M., Temmar M., Renard C., Choukroun G., Massy Z.A. (2015). The Addition of Vascular Calcification Scores to Traditional Risk Factors Improves Cardiovascular Risk Assessment in Patients with Chronic Kidney Disease. PLoS ONE.

[2] Ko W.-C., Choy C.-S., Lin W.-N., Chang S.-W., Liou J.-C., Tung T.-H., Hsieh C.-Y., Chang J.-F. (2018). Galectin-3 Interacts with Vascular Cell Adhesion Molecule-1 to Increase Cardiovascular Mortality in Hemodialysis Patients. J. Clin. Med..

[3] Chang J.-F., Liu S.-H., Lu K.-C., Ka S.-M., Hsieh C.-Y., Ho C.-T., Lin W.-N., Wen L.-L., Liou J.-C., Chang S.-W. (2020). Uremic Vascular Calcification Is Correlated with Oxidative Elastic Lamina Injury, Contractile Smooth Muscle Cell Loss, Osteogenesis, and Apoptosis: The Human Pathobiological Evidence. Front. Med. (Lausanne).

[4] Shroff R.C., Donald A.E., Hiorns M.P., Watson A., Feather S., Milford D., Ellins E.A., Storry C., Ridout D., Deanfield J. (2007). Mineral metabolism and vascular damage in children on dialysis. J. Am. Soc. Nephrol..

[5] Foley R.N., Parfrey P.S., Sarnak M.J. (1998). Clinical epidemiology of cardiovascular disease in chronic renal disease. Am. J. Kidney Dis..

[6] De Albuquerque Suassuna P.G., Sanders-Pinheiro H., De Paula R.B. (2018). Uremic Cardiomyopathy: A New Piece in the Chronic Kidney Disease-Mineral and Bone Disorder Puzzle. Front. Med..

[7] Cozzolino M., Ciceri P., Galassi A., Mangano M., Carugo S., Capelli I., Cianciolo G. (2019). The Key Role of Phosphate on Vascular Calcification. Toxins.

[8] Chang J.-F., Liang S.-S., Thanasekaran P., Chang H.-W., Wen L.-L., Chen C.-H., Liou J.-C., Yeh J.-C., Liu S.-H., Dai H.-M. (2018). Translational Medicine in Pulmonary-Renal Crosstalk: Therapeutic Targeting of p-Cresyl Sulfate Triggered Nonspecific ROS and Chemoattractants in Dyspneic Patients with Uremic Lung Injury. J. Clin. Med..

[9] Muteliefu G., Enomoto A., Jiang P., Takahashi M., Niwa T. (2009). Indoxyl sulphate induces oxidative stress and the expression of osteoblast-specific proteins in vascular smooth muscle cells. Nephrol. Dial. Transplant..

[10] Vanholder R., Schepers E., Pletinck A., Nagler E.V., Glorieux G. (2014). The uremic toxicity of indoxyl sulfate and p-cresyl sulfate: A systematic review. J. Am. Soc. Nephrol. JASN.

[11] Hénaut L., Mary A., Chillon J.-M., Kamel S., Massy Z.A. (2018). The Impact of Uremic Toxins on Vascular Smooth Muscle Cell Function. Toxins.

[12] Hénaut L., Mentaverri R., Liabeuf S., Bargnoux A.S., Delanaye P., Cavalier É., Cristol J.P., Massy Z., Kamel S. (2015). Pathophysiological mechanisms of vascular calcification. Ann. Biol. Clin..

[13] Proudfoot D., Skepper J.N., Hegyi L., Bennett M.R., Shanahan C.M., Weissberg P.L. (2000). Apoptosis regulates human vascular calcification in vitro: Evidence for initiation of vascular calcification by apoptotic bodies. Circ. Res..

[14] Tanaka H., Iwasaki Y., Yamato H., Mori Y., Komaba H., Watanabe H., Maruyama T., Fukagawa M. (2013). p-Cresyl sulfate induces osteoblast dysfunction through activating JNK and p38 MAPK pathways. Bone.

[15] Wakamatsu T., Yamamoto S., Ito T., Sato Y., Matsuo K., Takahashi Y., Kaneko Y., Goto S., Kazama J.J., Gejyo F. (2018). Indoxyl Sulfate Promotes Macrophage IL-1β Production by Activating Aryl Hydrocarbon Receptor/NF-κ/MAPK Cascades, but the NLRP3 inflammasome Was Not Activated. Toxins.

[16] Shimizu H., Bolati D., Higashiyama Y., Nishijima F., Shimizu K., Niwa T. (2012). Indoxyl sulfate upregulates renal expression of MCP-1 via production of ROS and activation of NF-κB, p53, ERK, and JNK in proximal tubular cells. Life Sci..

[17] Wu Y., Han X., Wang L., Diao Z., Liu W. (2016). Indoxyl sulfate promotes vascular smooth muscle cell calcification via the JNK/Pit-1 pathway. Ren. Fail..

[18] Barreto F.C., Barreto D.V., Liabeuf S., Meert N., Glorieux G., Temmar M., Choukroun G., Vanholder R., Massy Z.A., European Uremic Toxin Work Group (2009). Serum indoxyl sulfate is associated with vascular disease and mortality in chronic kidney disease patients. Clin. J. Am. Soc. Nephrol..

[19] Adijiang A., Goto S., Uramoto S., Nishijima F., Niwa T. (2008). Indoxyl sulphate promotes aortic calcification with expression of osteoblast-specific proteins in hypertensive rats. Nephrol. Dial. Transplant..

[20] Durham A.L., Speer M.Y., Scatena M., Giachelli C.M., Shanahan C.M. (2018). Role of smooth muscle cells in vascular calcification: Implications in atherosclerosis and arterial stiffness. Cardiovasc. Res..

[21] Shroff R.C., McNair R., Skepper J.N., Figg N., Schurgers L.J., Deanfield J., Rees L., Shanahan C.M. (2010). Chronic mineral dysregulation promotes vascular smooth muscle cell adaptation and extracellular matrix calcification. J. Am. Soc. Nephrol. JASN.

[22] Berti D.A., Seger R. (2017). The Nuclear Translocation of ERK. Methods Mol. Biol..

[23] Opdebeeck B., Maudsley S., Azmi A., De Maré A., De Leger W., Meijers B., Verhulst A., Evenepoel P., D’Haese P.C., Neven E. (2019). Indoxyl Sulfate and p-Cresyl Sulfate Promote Vascular Calcification and Associate with Glucose Intolerance. J. Am. Soc. Nephrol. JASN.

[24] Hsieh C.Y., Li L.H., Rao Y.K., Ju T.C., Nai Y.S., Chen Y.W., Hua K.F. (2018). Mechanistic insight into the attenuation of gouty inflammation by Taiwanese green propolis via inhibition of the NLRP3 inflammasome. J. Cell. Physiol..

[25] Hua K.F., Chou J.C., Ka S.M., Tasi Y.L., Chen A., Wu S.H., Chiu H.W., Wong W.T., Wang Y.F., Tsai C.L. (2015). Cyclooxygenase-2 regulates NLRP3 inflammasome-derived IL-1beta production. J. Cell. Physiol..

[26] Yeh J.C., Wu C.C., Choy C.S., Chang S.W., Liou J.C., Chen K.S., Tung T.H., Lin W.N., Hsieh C.Y., Ho C.T. (2018). Non-Hepatic Alkaline Phosphatase, hs-CRP and Progression of Vertebral Fracture in Patients with Rheumatoid Arthritis: A Population-Based Longitudinal Study. J. Clin. Med..

[27] Chang J.F., Feng Y.F., Peng Y.S., Hsu S.P., Pai M.F., Chen H.Y., Wu H.Y., Yang J.Y. (2014). Combined alkaline phosphatase and phosphorus levels as a predictor of mortality in maintenance hemodialysis patients. Medicine.

[28] Chang J.F., Chou Y.S., Wu C.C., Chen P.C., Ko W.C., Liou J.C., Hsieh C.Y., Lin W.N., Wen L.L., Chang S.W. (2020). A Joint Evaluation of Neurohormone Vasopressin-Neurophysin II-Copeptin and Aortic Arch Calcification on Mortality Risks in Hemodialysis Patients. Front. Med..

